# *Acidithiobacillus ferrianus* sp. nov.: an ancestral extremely acidophilic and facultatively anaerobic chemolithoautotroph

**DOI:** 10.1007/s00792-020-01157-1

**Published:** 2020-01-24

**Authors:** Paul R. Norris, Carmen Falagán, Ana Moya-Beltrán, Matías Castro, Raquel Quatrini, D. Barrie Johnson

**Affiliations:** 1 School of Biological Sciences, University of Warwick, Coventry, UK; 2 Environment and Sustainability Institute and Camborne School of Mines, University of Exeter, Penryn, UK; 3 School of Natural Sciences, Bangor University, Bangor, UK; 4 Fundación Ciencia y Vida, Santiago, Chile; 5 Universidad San Sebastian, Santiago, Chile; 6 Facultad de Ciencias de la Vida, Universidad Andres Bello, Santiago, Chile; 7 Millennium Nucleus in the Biology of Intestinal Microbiota, Santiago, Chile; 8 Facultad de Ingeniería y Tecnología, Universidad San Sebastián, Concepción, Chile; 9 Present Address: College of Engineering, Maths and Physical Sciences, University of Exeter, Penryn Campus, Penryn, Cornwall TR10 9FE UK

**Keywords:** *Acidithiobacillus ferrianus*, Ferrous iron oxidation, Ferric iron reduction, Hydrogen oxidation, Sulfur oxidation

## Abstract

Strain MG, isolated from an acidic pond sediment on the island of Milos (Greece), is proposed as a novel species of ferrous iron- and sulfur-oxidizing *Acidithiobacillus*. Currently, four of the eight validated species of this genus oxidize ferrous iron, and strain MG shares many key characteristics with these four, including the capacities for catalyzing the oxidative dissolution of pyrite and for anaerobic growth via ferric iron respiration. Strain MG also grows aerobically on hydrogen and anaerobically on hydrogen coupled to ferric iron reduction. While the 16S rRNA genes of the iron-oxidizing *Acidi-thiobacillus* species (and strain MG) are located in a distinct phylogenetic clade and are closely related (98–99% 16S rRNA gene identity), genomic relatedness indexes (ANI/dDDH) revealed strong genomic divergence between strain MG and all sequenced type strains of the taxon, and placed MG as the first cultured representative of an ancestral phylotype of iron oxidizing acidithiobacilli. Strain MG is proposed as a novel species, *Acidithiobacillus ferrianus* sp. nov. The type strain is MG^T^ (= DSM 107098^T^ = JCM 33084^T^). Similar strains have been found as isolates or indicated by cloned 16S rRNA genes from several mineral sulfide mine sites.

**Electronic supplementary material:**

The online version of this article (10.1007/s00792-020-01157-1) contains supplementary material, which is available to authorized users.

## Introduction

The genus *Acidithiobacillus* [phylum *Proteobacteria*, class *Acidithiobacillia* (Kelly and Wood 2000; Williams and Kelly 2013] currently includes eight validated species, which are found typically in low pH environments such as waste dumps at metal and coal mines and acidic waters draining from mine-impacted sites. All of these species can grow autotrophically using zero-valent (elemental) sulfur and sulfide, and sulfur-oxyanions that are more reduced than sulfate, as electron donors. Four of the species can oxidize ferrous iron: *A.* *ferrivorans* (Hallberg et al. 2010), *A.* *ferridurans* (Hedrich and Johnson 2013a), *A.* *ferriphilus* (Falagán and Johnson 2016) and the most studied iron-oxidizing species, *A.* *ferrooxidans* (Kelly and Wood 2000). Although ferric iron reduction may be observed in sulfur-containing cultures of all *Acidithiobacillus* spp., even under aerobic conditions, growth by ferric iron respiration appears to be restricted to those species that also oxidize iron (Hallberg et al. 2001; Johnson et al. 2017). Some acidithiobacilli can also use hydrogen as sole electron donor, but while this appears to be a common trait for all strains of some species (*A. ferrooxidans* and *A. ferridurans*) this is not the case for strains of other species (*A. ferrivorans, A. thiooxidans* and *A. caldus*) and has not been observed in any strain of *A. ferriphilus* (Hedrich and Johnson 2013b; Falagán and Johnson 2016).

The four currently-validated species of iron-oxidizing *Acidithiobacillus* share 98–99% identity of their 16S rRNA gene sequences but different species designations have been confirmed by comparison of additional marker genes (Amouric et al. 2011), MLSA-based phylogenies and oligotyping analysis (Nuñez et al. 2017). Several additional phylotypes have been identified among acidithiobacilli isolates and 16S rRNA gene sequence clones (Nuñez et al. 2017), a number of which currently lack cultured representatives and diagnostic phenotypic properties.

Here we describe strain MG, isolated from an acidic pond on the island of Milos (Greece), which represents a novel, ancestral phylotype of iron-oxidizing acidithiobacilli (phylotype 3A of Nuñez et al. 2017) and we present chemotaxonomic and genomic taxonomy data to support its recognition as a fifth iron oxidizing species, *A. ferrianus* MG^T^.

## Materials and methods

### Growth conditions

The liquid medium for growth with ferrous iron as substrate contained (g 1^−1^) (NH_4_)_2_SO_4_, (0.4), MgSO_4_·7H_2_O (0.5), K_2_HPO_4_ (0.2) and FeSO_4_·7H_2_O (13.9 g 1^−1^; equivalent to 50 mM ferrous iron), adjusted to pH 1.7 with H_2_SO_4_. Basal salts solutions were sterilized at 120 °C for 20 min, and ferrous sulfate solutions by filtering through 0.2 µm (pore size) membrane filters. For growth with sulfur (sterilized by Tyndallization), FeSO_4_.7H_2_O was lowered to 10 mg 1^−1^ (0.18 mM) sulfur powder (5 g l^−1^) added and pH adjusted to pH 3. Biomass particle analysis used a CellFacts Particle Size Analyzer (CellFacts Instruments, Coventry, UK). The ferrous iron medium was solidified with Phytagel (0.4%, w/v) for initial single colony isolation. Further growth studies with solid media used ferrous iron overlay plates (Johnson and Hallberg 2007). For solid medium anaerobic growth, oxygen was removed from sealed incubation jars containing activated carbon (AnaeroGen system, Fisher, U.K).

### Phenotype and chemotaxonomy observations

For scanning electron microscopy, cells were grown in basal salts/trace elements medium (Ñancucheo et al. 2016) containing sulfur as sole electron donor at 30 °C. Sulfur coupons were prepared as described by Castro et al. (2015). Samples were critical point dried, coated with gold and observed with a LEO 1420VP scanning electron microscope. For transmission electron microscopy, cells were grown aerobically at 30 °C to mid-exponential phase in basal salts/trace elements medium containing 2.5 mM potassium tetrathionate. Planktonic cells were harvested and fixed in 4% paraformaldehyde overnight. Samples were loaded onto a collodion-coated copper grid and stained with 1% (w/v) uranyl acetate. Samples were observed using a Philips Tecnai 12 (Biotwin) transmission electron microscope.

Strain MG was grown aerobically at 30 °C with hydrogen as substrate to provide biomass for analysis of fatty acids, polar lipids and respiratory quinones biomass at the DSMZ (*Deutsche Sammlung von Mikroorganismen und Zellkulturen*, Braunschweig, Germany). Chromosomal base composition was determined by thermal denaturation (Norris et al. 1996).

### Genome sequencing, molecular and phylogenetic analysis

Total DNA was extracted from strain MG following lysozyme treatment of cells grown on hydrogen. The genome was sequenced using paired-end libraries with insert size of ~ 460 bp (Nextera DNA Sample Preparation kit) and Illumina Hiseq sequencing technology (CD-Genomics, US). Reads were processed and assembled as described by Castro et al. (2017). The final draft assembly contained 90 contigs (N50 222, 906) and an average depth coverage of 33.42-fold adding up ~ 3.2 Mbp in total. This whole-genome shotgun project has been deposited at GenBank under the accession number WNJL00000000. The version described in this paper is version WNJL01000000. The average nucleotide identities between the draft genome and those of the reference type strains of *Acidithiobacillus* spp. were calculated using a Python module implemented by Goris et al. (2007) and available at http://widdowquinn.github.io/pyani/PYANI. The dDDH values between strains were calculated using the Genome-to-Genome Distance Calculator (GGDC) web server (Meier-Kolthoff et al. 2013). Analysis of the 16S rRNA gene used the complete sequence (MN733279) retrieved from the genome using BARNAP (BAsic Rapid Ribosomal RNA Predictor version 0.9-dev). This gene was 100% identical to the 16S rRNA gene sequence originally deposited in GenBank (MG062778) after the initial single colony isolation. The phylogeny of strain MG was assessed from the small subunit ribosomal RNA gene sequences alignment (MAFFT v7.229) (Katoh and Standley 2013), after manual trimming and masking (> 50%). Phylogenetic trees were reconstructed with two different algorithms (Neighbor-Joining and Maximum-Likelihood; Falagán et al. 2019) and their topologies compared. The consensus tree was constructed using PHYLIP (Shimada and Nishida 2017).

## Results and discussion

### Isolation and distribution

Strain MG was isolated from sediment of an acidic pond (approximately 12 m^2^ surface area) close to the geothermal site at Kalamos on the South coast of the island of Milos, Greece (Supplementary Fig. S1). The water was at ambient temperature, lightly coloured green from growth of unicellular algae, and was pH 3.5. There were patches of land surface in the area that were more acidic at about pH 2. The water was essentially chloride-free and contained 5 mg l^−1^ ferrous iron. Ferrous iron oxidation was observed in a ferrous iron enrichment culture (pH 2) of sediment from the pond margin and ferric iron-encrusted colonies were readily obtained on Phytagel-solidified medium containing 25 mM ferrous iron at pH 2. All colonies were of similar appearance and size. Identical 16S rRNA gene sequences were obtained from the two of the colonies that were examined. Two closely related iron-oxidizing acidithiobacilli have been isolated from sites in China and similar bacteria from other mine-impacted environments in China and the USA have been indicated by highly similar 16S rRNA gene sequences (between 98 and 99% identity to that of strain MG; Table 1). These isolates, clones and isolate MG could represent strains of the same species.

**Table 1:** Origins of isolates and clones with 16S rRNA gene sequences which have greater than 99.6% identity to that of strain MG

		GenBank acc. no.
*Isolates*		
LMT1	Mine tailings, Lechang, China; pH 1.9 (Tan et al. 2008)	AM502930
Ish-01	Soil, China	EU158322
*Clones*		
Fe-K6-C12	Mine tailings, Klondykee Mill, USA; pH 5.7 (Méndez et al. 2008)	EF612430
K6-C79	Mine tailings, Klondykee Mill, USA; pH 5.7 (Méndez et al. 2008)	EF612421
AMD-A14	Jinkouling tailings pond, Tongling, China; pH 2.65 (Yang et al. 2014)	KC620596
AMD-D35	Shuimuchong tailings pond, Tongling, China; pH 2.1 (Yang et al. 2014)	KC620779
G28	Yunfu sulfide mine, Guangdong, China; pH 2.5 (He et al. 2007)	DQ480479
X18	Copper sulfide ore bioleaching heap, China	FJ268717

### Phylogeny and genomic comparisons

**Fig. 1:**
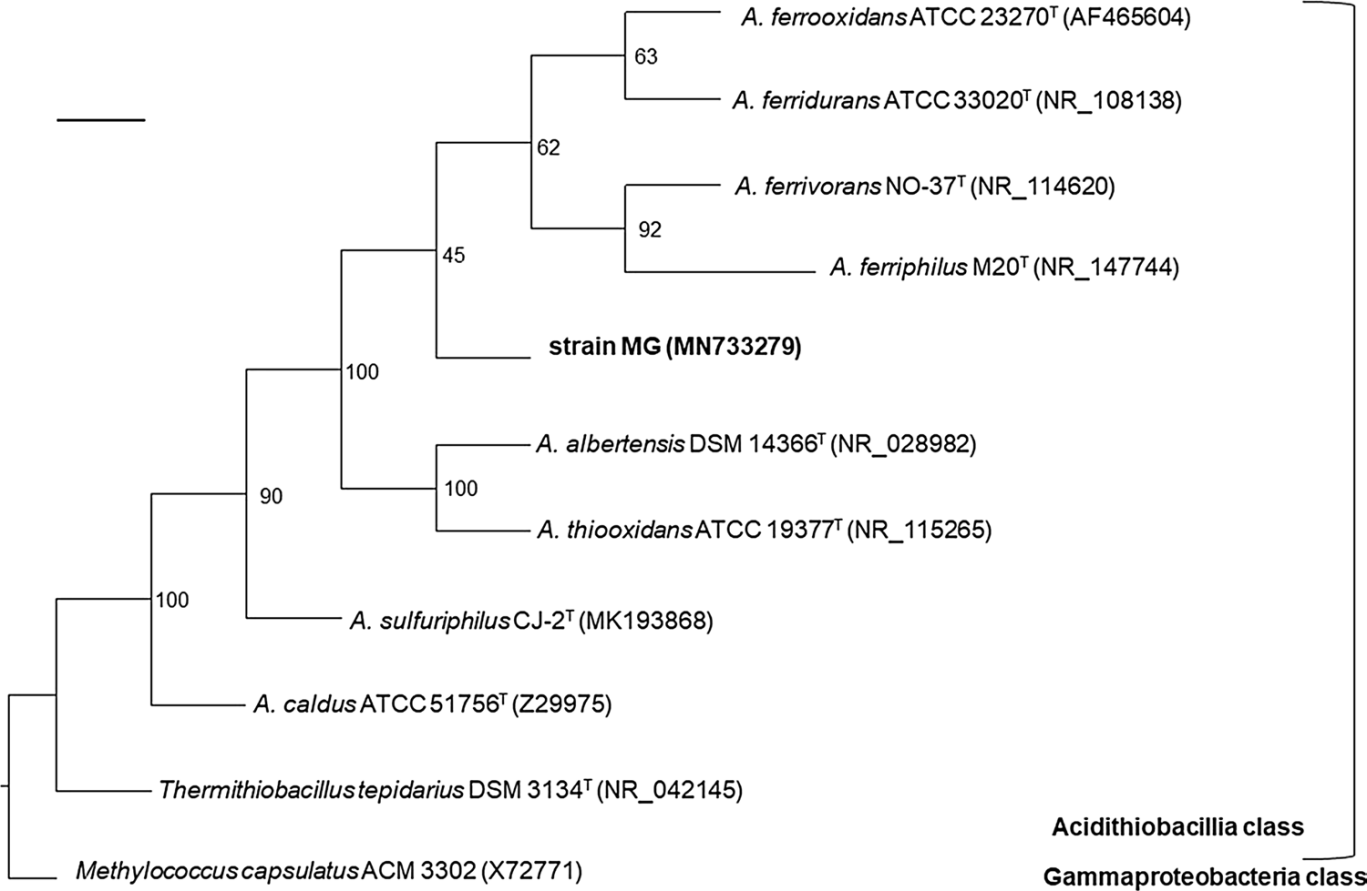
Consensus phylogenetic tree derived from the 16S rRNA gene sequences showing the relationship of strain MG with the type strains of the species with valid names of the genus *Acidithiobacillus* and *Thermithiobacillus*, the only other known genus in the Class. The gammaproteobacterium *Methylococcus capsulatus* ACM 3302 was used as outgroup. Bootstrap values are indicated at the respective nodes in the consensus tree derived from ML, NJ and BI phylogenetic treeing algorithms. Scale bar: 0.07% sequence divergence

Phylogenetic analysis of the 16S rRNA gene sequences placed strain MG outside the clade grouping the other iron-oxidizing members of the genus (Fig. 1) suggesting it represents an ancestral phylotype of iron-oxidizing acidithiobacilli, phylotype 3A of Nuñez et al. (2017). Limited disagreement in topology was observed between trees built using Neighbour-Joining and Maximum-Likelihood methods (Supplementary Fig. S2). Comparison of the sequences of strain MG and type strains of the genus *Acidithiobacillus* revealed identity differences (Table 2) which fall above the proposed species level cut-off value of > 1.3% (Stackebrandt and Ebers 2006), except in the case of *A.* *ferriphilus* and *A.* *ferridurans* for which the difference is marginally within this cut-off value. Four distinct clades comprising isolates of the four previously named ferrous iron-oxidizing species and a fifth for strain MG were also seen in re-construction of phylogenetic trees of two (*recA* and *atpD*) marker genes previously used (Amouric et al. 2011) to illustrate the genetic diversity of *A. ferrooxidans*-like isolates (Supplementary Fig. S3). Genomic relatedness indices further supported differentiation of strain MG from the named iron-oxidizing *Acidithiobacillus* species. Pairwise comparisons between strain MG and the available reference genomes (Table 3) gave values, in both cases, well below the established thresholds used for prokaryotic species delimitation. A DNA:DNA hybridization of about 35% for strain MG against *A.* *ferrooxidans* (using digoxigenin nucleic acid labelling and chemiluminescence detection) suggested a similar divergence between the type strains of these species to those of the type strains of *A.* *ferrooxidans* from *A.* *ferrivorans* and *A.* *ferriphilus* (L. Laigle and P. Norris, unpublished data).

**Table 2:** Identities of the strain MG 16S rRNA gene to those of *Acidithiobacillus* type strains (from alignment of the regions between nucleotide coordinates 31 and 1488; sequences recovered from GenBank(nr) and RefSeq databases)

Accession number	Species	% Identity
MN733279^a^; MG062778	Strain MG	100.00
NR_147744	*Acidithiobacillus ferriphilus*^T^	98.87
NR_108138	*Acidithiobacillus ferridurans*^T^	98.86
AF465604	*Acidithiobacillus ferrooxidans*^T^	98.56
NR_114620	*Acidithiobacillus ferrivorans*^T^	98.31
NR_028982	*Acidithiobacillus albertensis*^T^	98.13
NR_115265	*Acidithiobacillus thiooxidans*^T^	97.11
KX426303	*Acidithiobacillus sulfuriphilus*^T^	97.14
Z29975	*Acidithiobacillus caldus*^T^	95.63

^a^16S rRNA gene sequence retrieved from the MG strain genome using BARNAP (BAsic Rapid Ribosomal RNA Predictor version 0.9-dev)

**Table 3:** Genomic relatedness indexes (%) calculated between strain MG and acidithiobacilli reference strains

Accession no.	Strain	dDDH^a^	ANIb^b^	ANIm^b^
WNJL01	Strain MG DSM 107098^T^	100.00	100.00	100.00
NC_015942	*A. ferrivorans* SS3	25.00	81.28	85.57
LVXZ01	*A. ferriphilus* BY0502	25.40	81.07	85.56
NC_011761	*A. ferro-oxidans* ATCC 23270^T^	24.60	80.93	85.57
NZ_AP018795	*A.* *ferridurans* JCM18981	24.30	80.37	85.23
RIZI01	*A. sulfuriphilus* DSM 105150^T^	21.80	74.75	86.20
AF0H01	*A. thiooxidans* ATCC 19377^T^	19.80	74.24	84.82
MOAD01	*A. albertensis* DSM 14366^T^	20.20	74.27	83.62
CO005986	*A. caldus* ATCC 51756^T^	19.00	72.81	86.56
AUIS01	*T. tepidarius* DSM 3134^T^	19.30	71.97	83.12

^a^DNA–DNA hybridization species-level cutoff: > 70 (Meier-Kolthoff et al. 2013)

^b^Average Nucleotide Identity using BLAST (ANIb) and MUMmer (ANIm) species-level cutoff: > 95.9 (Goris et al. 2007)

### Phenotypic characteristics

**Fig. 2:**
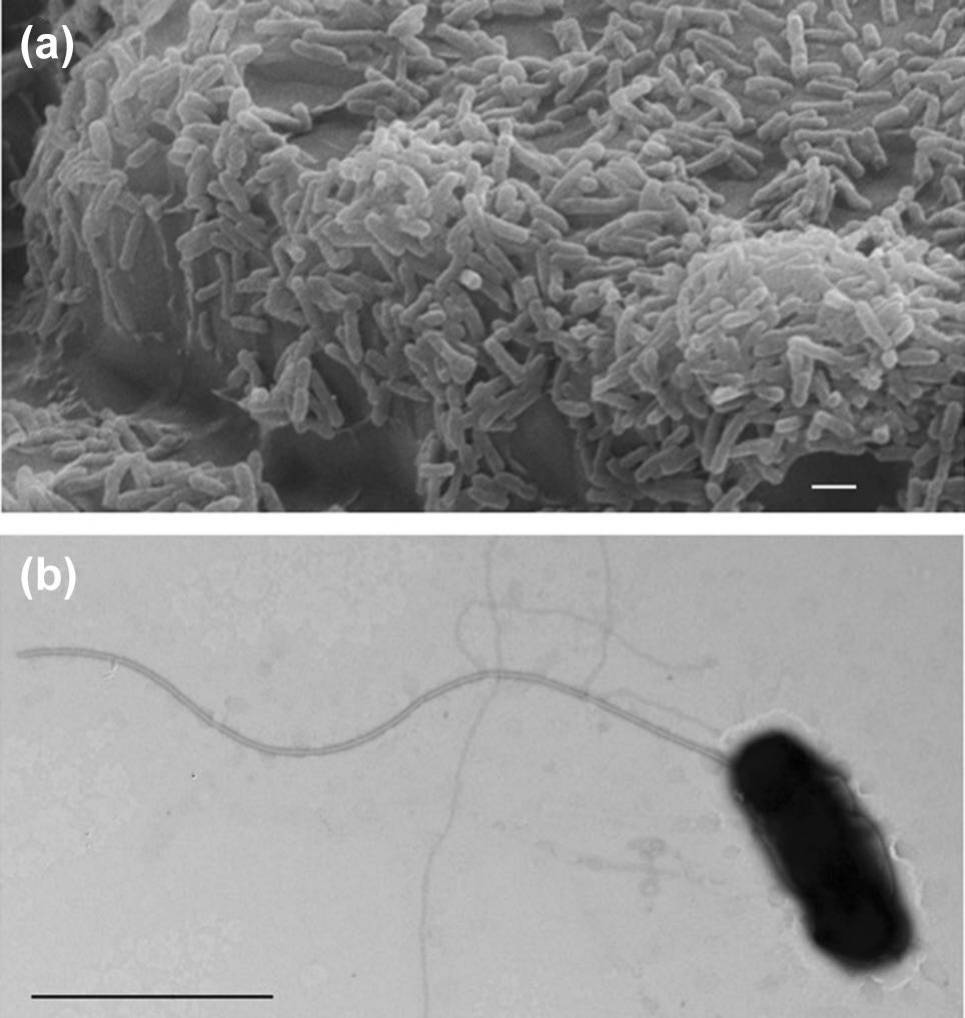
Representative images of strain MG under substrate-attached and planktonic growth conditions. **a** Scanning electron microscopy (SEM) of strain MG attached on sulfur coupons during biofilm development. Scale bar, 1 µm. **b** Transmission electron microscopy (TEM) of a single motile cell showing a monopolar flagellum. Scale bar, 1 µm

Scanning electron microscopy showed attached cells and copious biofilms of sulfur-grown strain MG (Fig. 2a). A polar flagellum was observed during growth with tetrathionate (Fig. 2b). Flagellated cells swimming in tight groups were also observed, suggesting that strain MG has the capacity to swarm (data not shown). Motility was also observed during growth on ferrous iron.

**Fig. 3:**
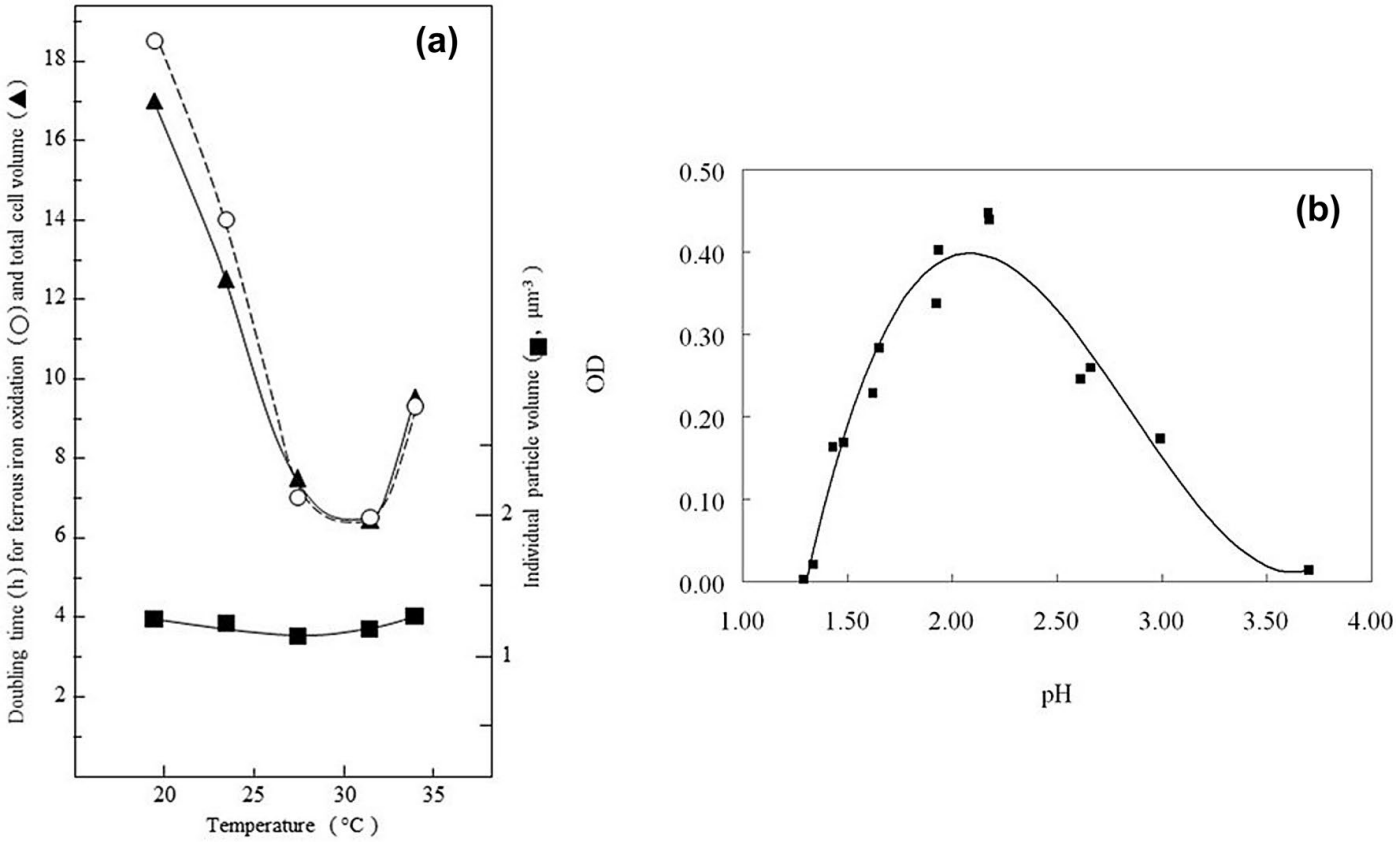
Effect of temperature on growth of strain MG with ferrous iron as electron donor (**a**), and of pH with hydrogen as electron donor (**b**)

In liquid media containing ferrous iron as sole electron donor, strain MG grew with a culture doubling time of between 6 and 7 h at an optimum temperature between 28 and 30 °C (Fig. 3a). Particle size analysis indicated little size change or aggregation of single and dividing cells over a temperature range of about 20 °C to at least 32 °C (Fig. 3a). Growth on ferrous iron was slightly slower at an initial culture medium pH of 2.0 than at pH 1.7 (data not shown). The mechanism of ferrous iron oxidation by strain MG could involve the *rus* operon, which is found in all of the iron-oxidizing acidithiobacilli, with key electron transport proteins rusticyanin and Cyc2 of strain MG sharing 94 and 85% amino acid identity respectively with those of *A.* *ferrooxidans*^T^ (Norris et al. 2018).

The optimum pH for aerobic growth with hydrogen as sole substrate was *circa*. pH 2.2 with incubation under H_2_/CO_2_-enriched air (Fig. 3b), where high cell densities (> 10^9^ cells m l^−1^) were reached in liquid medium which contained basal salts, trace elements and 25 μM ferrous iron. Addition of yeast extract (0.02% w/v) or glycerol (5 mM) to ferrous iron liquid medium did not result in any increase in cell numbers of strain MG in oxidized cultures (data not shown) suggesting that, like other *Acidithiobacillus* spp., it is an obligate autotroph.

**Fig. 4:**
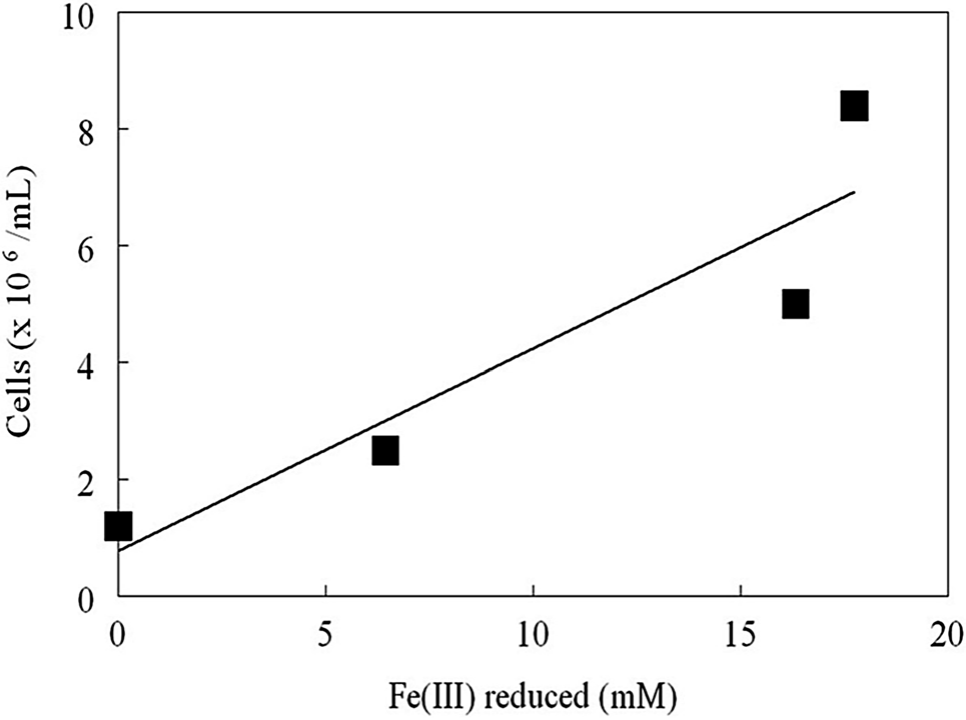
Correlation between cell numbers of strain MG and ferric iron reduced with hydrogen as electron donor and ferric iron as electron acceptor

Ferric iron-encrusted colonies of strain MG grew on ferrous iron overlay plates that were incubated under H_2_/CO_2_-enriched air. The morphology of colonies was similar initially to that of those incubated under air but, with more protracted (2–3 weeks) incubation, the colonies developed off-white gelatinous secondary growths that eventually occluded the iron-encrusted zones. When the secondary growths were re-streaked onto fresh plates and incubated under H_2_/CO_2_-enriched air, most colonies did not accumulate ferric iron, though a minority appeared to revert to oxidizing iron (Supplementary Fig. S4a). Two distinct non-iron-encrusted colony morphologies were found, one smooth and the other larger and crustose (Supplementary Fig. S4b). These morphologies were retained when single colonies were sub-cultured. 16S rRNA genes from the three colony variants (ferric iron-encrusted, off-white smooth and crustose forms) were all identical to those from ferrous iron- and sulfur-grown strain MG. Growth with hydrogen as sole electron donor also occurred anaerobically in the presence of ferric iron, with cell numbers correlating with the amount of iron reduced demonstrating that in common with other iron-oxidizing acidithiobacilli, strain MG is a facultative anaerobe (Fig. 4).

**Fig. 5:**
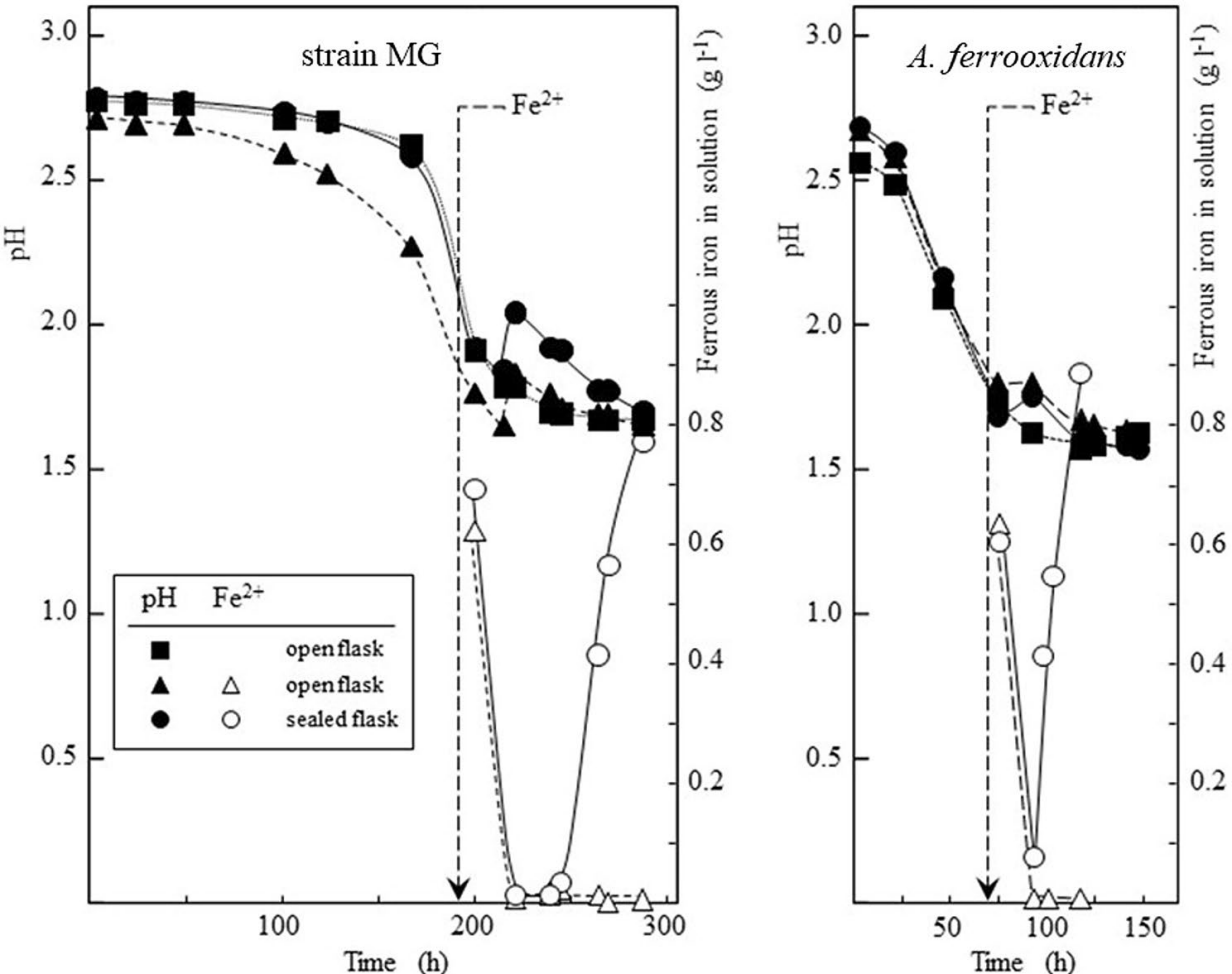
Growth of strain MG and *A.* *ferrooxidans* ATCC 23270^T^ at 30 °C with elemental sulfur, showing initial oxidation of ferrous iron (0.9 g l^−1^ added at the times indicated by arrows) followed by reduction of produced ferric iron when oxygen became depleted in sealed flasks

Autotrophic growth of strain MG with sulfur as electron donor could also be coupled to either oxygen or ferric iron as electron acceptors. Strain MG appeared to have a longer lag phase on serial sub-culturing with sulfur than *A. ferrooxidans* (Fig. 5). Both species showed a similar response to oxygen depletion after addition of ferrous iron (as ferrous sulfate) to cells growing on sulfur. Acid-consuming ferrous iron oxidation was followed by reduction of the ferric iron produced and further acidification when oxygen became depleted in sealed flasks (Fig. 5), suggesting strain MG grew anaerobically using ferric iron to oxidize sulfur, as shown for *A.* *ferrooxidans* (Pronk et al. 1992), *A.* *ferridurans* (Hedrich and Johnson 2013a, b), *A.* *ferrivorans* (Hallberg et al. 2010) and *A.* *ferriphilus* (Falagán and Johnson 2016).

In common with other ferrous iron-oxidizing acidi-thiobacilli, strain MG catalyzed the oxidative dissolution of pyrite with concomitant increases in soluble iron and redox potential during growth (Supplementary Fig. S5). Even without prior adaptation, strain MG tolerated high concentrations of transition metals in solution but was highly sensitive to molybdenum (which exists predominantly as non-dissociated molybdic acid at pH 2), as is seen with other iron-oxidizing acidophiles (Supplementary Table S1). It was also found to be highly osmotolerant, growing in media containing > 1 M magnesium sulfate, but it was more sensitive to (sodium) chloride, with no growth observed (at pH 2) with > 0.3 M NaCl.

### Chemotaxonomic analyses

The major fatty acids of strain MG, C_18:1_*ω* 7c, C_16:1_*ω* 7c/C_16:1_*ω* 6 and C_16:0_, were similar in relative abundance to those reported for other iron-oxidizing acidithiobacilli (Table 4; no published data are available for *A.* *ferrivorans*). The major polar lipids of strain MG were aminolipid, phosphatidylglycerol and phosphotidylethanolamine, and the major quinone present (95%) was Q8 (as also reported for *A.* *ferridurans* (Hedrich and Johnson 2013a, b) and *A.* *ferriphilus* (Falagán and Johnson 2016)) with smaller amounts of Q7 (5%). Summed features represent groups of two or three fatty acids that could not be separated by GLC with the MIDI system. Summed feature 1 contains iso-C_15:1_ and/or iso-C_13:0_ 3-OH.; summed feature 2 contains C_14:0_ 3-OH and/or iso-C_16:1_; summed feature 3 contains C_16:1_ω 7c, C_16:1_ω6c and/or iso-C_15:0_ 2-OH; summed feature 8 contains C_18:1_ω 7c and/or C_18:1_ω 6c.

**Table 4:** Cellular fatty acids (shown as percentage values) of strain MG grown on hydrogen at pH 2 and 30 °C and comparison with values reported for the type strains of iron-oxidizing *Acididthiobacillus* spp. (Falagán and Johnson 2016). Summed features represent groups of two or three fatty acids that could not be separated by GLC with the MIDI system. *Summed feature 1 contains iso-C_15:1_ and/or iso-C_13:0_ 3-OH.; summed feature 2 contains C_14:0_ 3-OH and/or iso-C_16:1_; summed feature 3 contains C_16:1_*ω* 7c, C_16:1_*ω* 6c and/or iso-C_15:0_ 2-OH; summed feature 8 contains C_18:1_*ω* 7c and/or C_18:1_*ω* 6c

Fatty acid	Strain MG	*A. ferrooxidans*^T^	*A. ferridurans*^T^	*A. ferriphilus*^T^
C_12:0_	5.7	8	6.6	5.7
C_13:_ AT12-13	–	11	–	0.4
C_14:0_	0.4	–	0.3	0.2
C_15:0_	–	–	0.7	–
C_15:0_ 3-OH	–	–	0.5	–
C_16:0_	17.6	18	15.6	7.5
C_16:0_ 2-OH	2.1	–	1.2	0.5
C_16:0_ 3-OH	2.4	–	0.9	2.7
C_16:1_	–	21	–	–
C_16:1_*ω* 5c	–	–	–	0.4
C_17:0_	0.9	6	1.9	0.5
C_17:0_ cyclo	3.1	–	6.7	–
C_17:0_ 2-OH	0.3	–	–	0.1
C_17:1_*ω* 6c	–	–	–	0.4
C_17:1_*ω* 8c	0.4	0.5	0.7	0.6
C_18:0_	0.8	0.5	1.5	0.9
C_18:0_ 2-OH	0.2	–	–	0.5
C_18:0_ 3-OH	0.4	–	–	0.1
C_18: 1_ ω5c	0.2	–	0.6	–
C_18:1_*ω* 7c	24.1	21.5	16.6	33.8
C_18: 1_ 2-OH	3.3	–	0.9	10.3
11 methyl C_18:1_*ω* 7c	–	–	0.3	–
C_19:0_ 10 methyl	0.6			1.0
C_19:0_ cyclo *ω* 8c	5.2	14.5	17.5	–
C_20:2_*ω* 6, 9c	–	–	0.4	–
Summed feature 1*	0.2	–	0.3	–
Summed feature 2*	9.0	–	9.9	10.14
Summed feature 3*	23.1	–	14.9	21.57
Summed feature 8*	24.1	–	–	–

The mean base composition of the chromosomal DNA of strain MG was determined as 58 mol% G + C by thermal denaturation and the draft genome contigs have an average of 58.2 mol% G + C. All previously described iron-oxidizing acidithiobacilli have DNA containing between 56 and 59 mol% G + C.

In summary, a species designation for strain MG is strongly supported by different pieces of evidence, including 16S rRNA and marker gene-based taxonomy, genomic taxonomy and chemotaxonomy. Overall genome relatedness indices derived from the available genome sequences of the taxon are in line with DNA:DNA hybridization values and provide strong evidence supporting the genomic divergence of strain MG and currently acknowledged type strains of the taxon. Of particular interest was the observation that strain MG is the first characterized representative of an ancestral phylotype of iron oxidizing acidithiobacilli. Further isolations will be required to elucidate physiological and phylogenetic variabilities of the novel species and reveal its wider geographical and ecological distribution.

### Description of *Acidithiobacillus ferrianus* sp. nov.

*Acidithiobacillus ferrianus* (*fer.ri.a’nus. L. neut. n. ferrum iron; L. masc. n. Ianus*, Roman god of gates and duality, often depicted with two opposite-facing heads); *N.L. masc. n. ferrianus*, referring to its ability both to oxidize and reduce iron.

Gram-negative, motile, flagellated, straight rods (1.2–2.5 μm in length) that do not form endospores. Forms ferric iron-stained colonies on acidic ferrous iron-containing solid media. Obligate chemolithoautotroph, capable of growth using ferrous iron, elemental sulfur or hydrogen as electron donors. Poor growth on tetrathionate. Facultative anaerobe, capable of coupling oxidation of ferrous iron, sulfur and hydrogen to reduction of molecular oxygen, and oxidation of sulfur and hydrogen to reduction of ferric iron. Mesophilic and acidophilic with optimum growth about pH 2 and 30 °C. The G + C content of the chromosomal DNA of the type strains is 58.2%. The type strain, *A. ferrianus* strain MG^T^ (= DSM 107098^T^, = JCM 33084^T^) was isolated from an acidic pond close to the geothermal site at Kalamos on the South coast of the island of Milos, Greece.

## Electronic supplementary material

Below is the link to the electronic supplementary material.

Supplementary material 1 (DOCX 1014 kb)

## Abbreviations

dDDH: In silico DNA–DNA hibridization
ANIb^2^: Average Nucleotide Identity using blast
ANIm^2^: Average Nucleotide Identity using MUMmer
