# Supplementary material for: Acidithiobacillus ferrianus sp. nov.: an ancestral extremely acidophilic and facultatively anaerobic chemolithoautotroph
Source: Extremophiles. 2020 Jan 24;24(2):329–37. doi: 10.1007/s00792-020-01157-1 (PMC7040056; doi:10.1007/s00792-020-01157-1)
Supplement: Supplementary file 1 — Supplementary material 1 (DOCX 1014 kb) [file 792_2020_1157_MOESM1_ESM.docx]

**Supplementary Information**

***Acidithiobacillus ferrianus* sp. nov.: an ancestral extremely acidophilic and facultatively anaerobic chemolithoautotroph**

Submitted to the journal ***Extremophiles***

Paul R. Norris^1†^, Carmen Falagán^2,3^, Ana Moya-Beltrán^4,5^, Matías Castro^4,6^, Raquel Quatrini^4,7^, D. Barrie Johnson^3*^

Author affiliations: ^1^School of Biological Sciences, University of Warwick, UK; ^2^ Environment and Sustainability Institute and Camborne School of Mines, University of Exeter, UK; ^3^School of Natural Sciences, Bangor University, Bangor, UK; ^4^Fundación Ciencia y Vida- Universidad San Sebastian, Santiago, Chile; ^5^Universidad Andrés Bello, Santiago, Chile; ^6^Facultad de Ingeniería y Tecnología, Universidad San Sebastián, Concepción, Chile; ^7^Millennium Nucleus in the Biology of Intestinal Microbiota, Santiago, Chile.

**Present address**: ^†^College of Engineering, Maths and Physical Sciences, University of Exeter, Penryn Campus, Penryn, UK.

*Correspondence: [d.b.johnson@bangor.ac.uk](mailto:d.b.johnson@bangor.ac.uk). Tel. +44 1248 382358; Fax +44 1248 370731

**Supplementary Table S1.** Tolerance of type strains of iron-oxidizing *Acidithiobacillus* spp. to some transition metals, magnesium, molybdenum and salt (NaCl), as maximum concentrations where growth was observed (MAX) and minimum concentrations where growth was completely inhibited (MIC). nr, not recorded

| **Metal/salt** | **Bacterium** | | | | | | | | | |
| --- | --- | --- | --- | --- | --- | --- | --- | --- | --- | --- |
|  | ***A. ferrianus*^T^** | | *A. ferrooxidans*^T^ | | *A. ferrivorans*^T^ | | *A. ferridurans*^T^ | | *A. ferriphilus*^T^ | |
|  | MAX | MIC | MAX | MIC | MAX | MIC | MAX | MIC | MAX | MIC |
| Co | 50 | 100 | nr | nr | nr | nr | nr | nr | 400 | 600 |
| Cu | 300 | 500 | 400 | 500 | <50 | nr | 200 | 300 | 300 | 500 |
| Fe(III) | >500 | nr | 200 | 400 | <100 | nr | 200 | 300 | 300 | 500 |
| Mg | >1000 | nr | 800 | 1000 | 800 | 1000 | 1000 | 1200 | 900 | 1000 |
| Mo | <0.1 | nr | 0.1 | 0.25 | <0.1 | nr | <0.1 | nr | <0.1 | nr |
| Ni | 50 | 100 | 100 | 200 | 200 | 300 | 200 | 300 | 300 | 500 |
| Zn | 700 | 800 | 800 | 1000 | 200 | 300 | 800 | 1000 | 700 | 800 |
| NaCl | 300 | 500 | 100 | 200 | 90 | 100 | 300 | 500 | 250 | 500 |

**References**

Falagán C, Johnson DB (2016) *Acidithiobacillus ferriphilus* sp. nov.: a facultatively anaerobic iron- and sulfur-metabolising extreme acidophile. Int J Syst Evol Microbiol 66:206-211.

Hallberg KB, González-Toril E, Johnson DB (2010) *Acidithiobacillus ferrivorans* sp. nov.; facultatively anaerobic, psychrotolerant, iron- and sulfur-oxidizing acidophiles isolated from metal mine-impacted environments. Extremophiles 14:9-19.

Hedrich S, Johnson DB (2013) *Acidithiobacillus ferridurans*, sp. nov.; an acidophilic iron-, sulfur and hydrogen-metabolizing chemolithotrophic

gammaproteobacterium. 63:4018-4025.


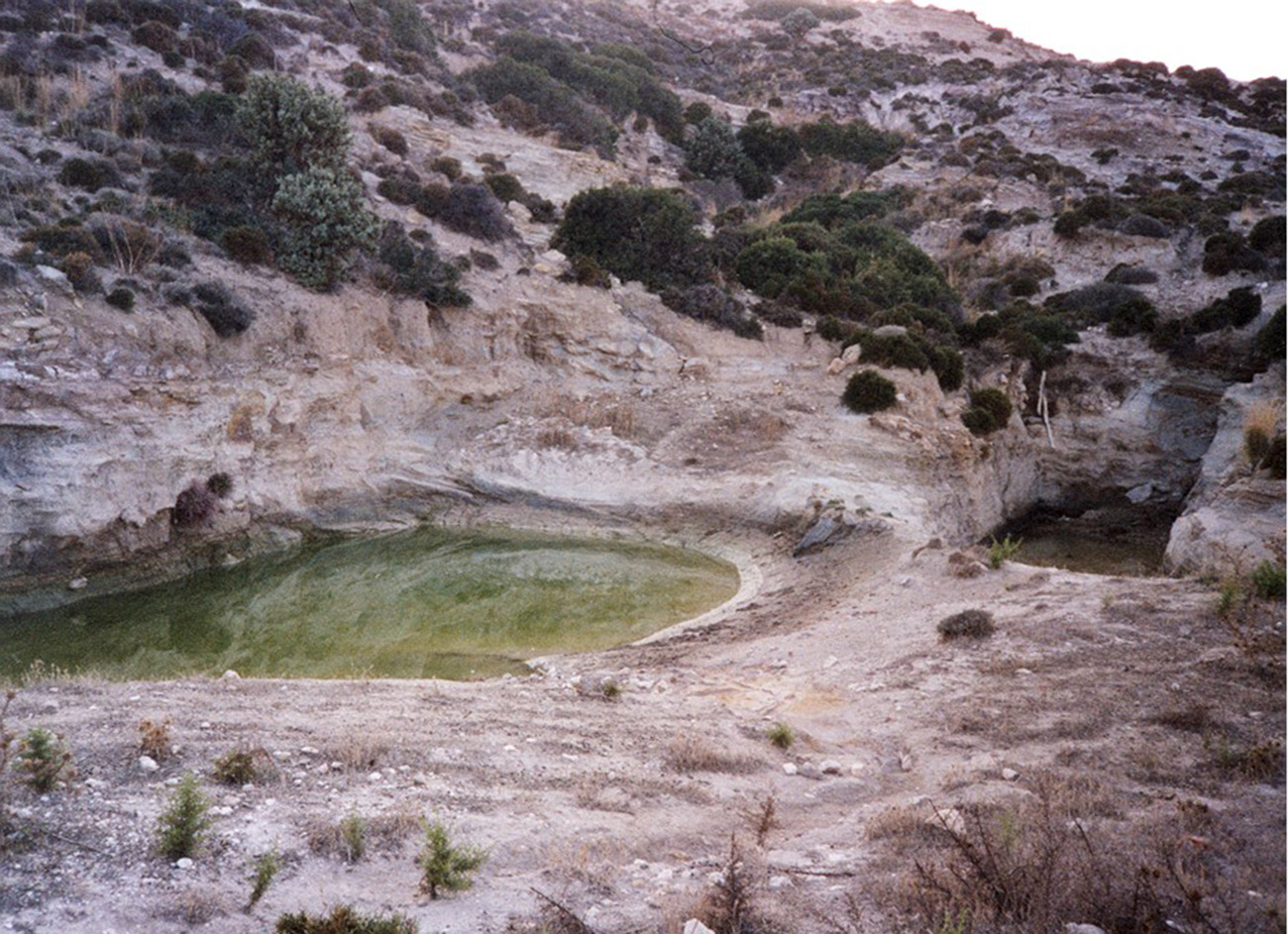


**Supplementary Fig. S1** Site of *A. ferrianus*^T^ isolation, an acidic pond (approximately 6m x 2m in area) close to the geothermal site at Kalamos on the South coast of the island of Milos, Greece


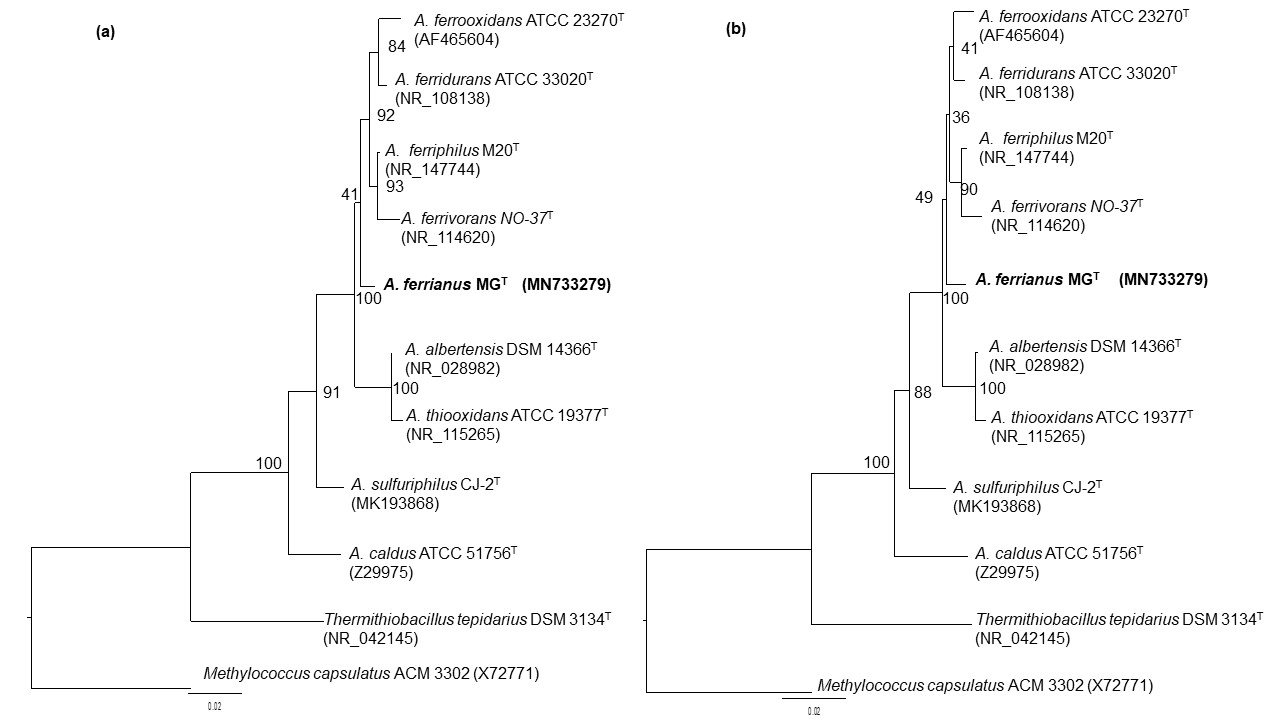


**Supplementary Fig. S2** 16S rRNA gene phylogenetic trees showing the relationship of the MG^T^ strain with the type strains of the *Acidithiobacillus (A.)* genus. *Methylococcus capsulatus* ACM 3302 (X72771) was used as outgroup. *Thermithiobacillus* (*T.*) *tepidarius* DSM 3134^T^, the type strain of the only other genus in the *Acidithiobacillaceae* family was also included for comparison. MAFTT v7.310 was used for sequences alignments, and the Trees built using (A) the Maximum-Likelihood algorithm with FastTree version 2.1.10 or (B) the Neighbor-Joining algorithm with Phylip version 3.698. Bootstrap values are indicated at the respective nodes, when different from 100%. Sequence divergence scale bar: 0.02 %


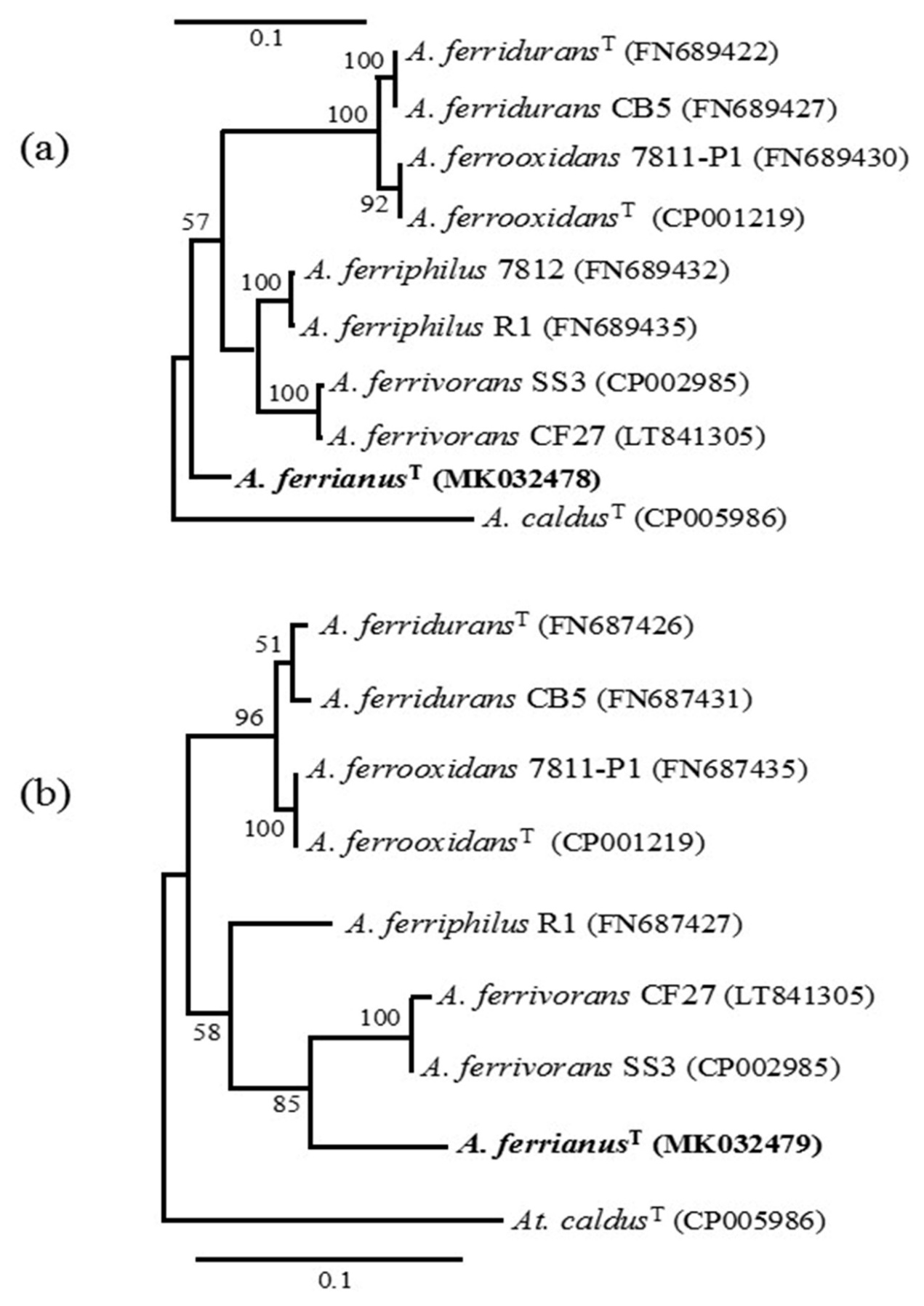


**Supplementary Fig. S3** Maximum likelihood phylogenetic trees for partial (a) *atpD* (475) and (b) *recA* (808 aligned positions) genes of ferrous iron-oxidizing *Acidithiobacillus* species and an *A. caldus* outgroup. GenBank accession numbers for each sequence are in parentheses


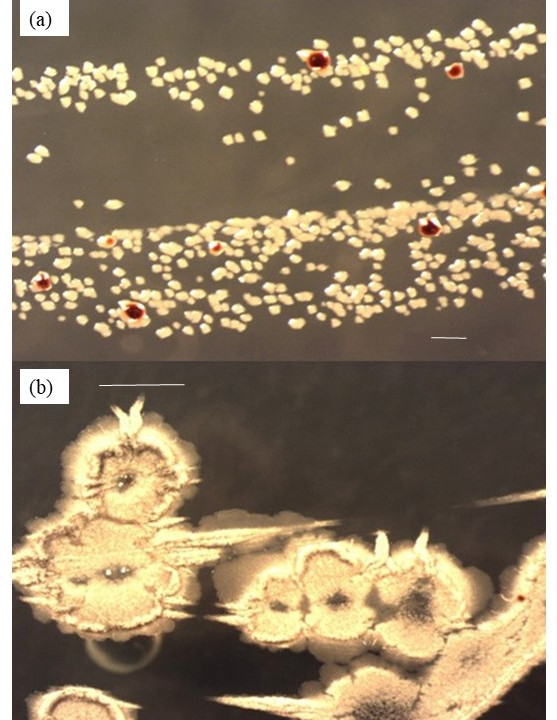


**Supplementary Fig. S4** Colonies of strain *A. ferrianus*^T^ grown on ferrous iron overlay solid medium incubated under H_2_/CO_2_-enriched air: (a) smooth unstained and ferric iron-stained colonies; (b) crustose colonies. Scale bars represent 5 mm


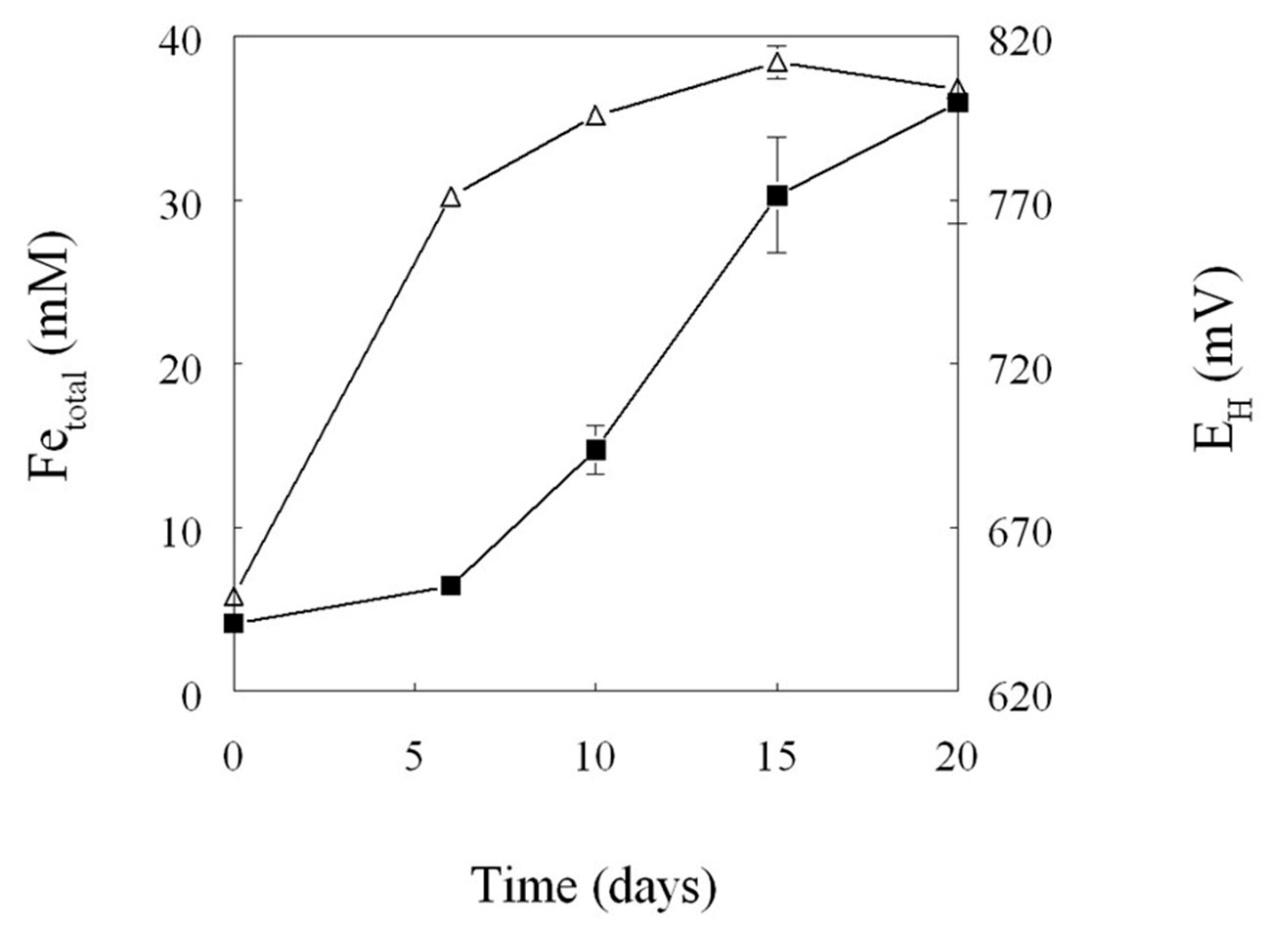


**Supplementary Fig. S5** Oxidative dissolution of pyrite (FeS_2_) by *A. ferrianus*^T^, showing changes in concentrations of total soluble iron (■) and culture redox potentials (Δ)
